# Integrative multi-omics analysis reveals gut-skin axis mechanisms and novel therapeutic target *GALE* in atopic dermatitis

**DOI:** 10.1128/msystems.01403-25

**Published:** 2025-12-05

**Authors:** Fang Cao, AoNan Liu, Jiaoyang Tong, Cui Guo, Hui Zhang, Yaobin Pang, Kexin Tang, Qianying Yu, Jing Guo

**Affiliations:** 1 Chengdu University of Traditional Chinese Medicine 118385, Chengdu, Sichuan, China; 2 Shanghai Jiao Tong University School of Medicine 56694, Shanghai, China; 3 Department of Rheumatology and Immunology, First Affiliated Hospital of Army Military Medical University 388288 , Chongqing, China; LifeMine Therapeutics, Cambridge, Massachusetts, USA

**Keywords:** gut-skin axis, gut microbiota, atopic dermatitis, bioinformatics, computational pharmacology

## Abstract

The gut-skin axis represents a critical but poorly understood pathway in atopic dermatitis (AD) pathogenesis. This study aimed to establish causal relationships between gut microbiota and AD risk while identifying key molecular bridges and therapeutic targets. We integrated multiple analytical approaches, including single-cell RNA sequencing analysis of skin biopsies from five AD patients and four healthy controls, intercellular communication network analysis, pseudotime trajectory inference, reverse drug prediction, molecular docking, and molecular dynamics simulations. Analysis revealed increased keratinocyte heterogeneity and enhanced immune cell communication in atopic dermatitis (AD) samples. Intersection analysis between gut microbial metabolite-associated genes and skin pathology-related genes identified seven key bridging genes (*AKR1C2*, *GALE*, *GGH*, *NR4A1*, *PLA2G4B*, *TYMS*). Functional annotation indicated that these genes are primarily involved in vitamin precursor metabolism, suggesting that the *Eubacterium eligens* group influences AD pathogenesis mainly through vitamin precursor-mediated pathways that regulate systemic immune responses. Pseudotime trajectory analysis demonstrated dynamic temporal gene expression patterns during disease progression. Molecular docking revealed an unexpectedly high-affinity binding between methotrexate and *GALE* (binding energy = −10.4 kcal/mol), which exceeded its binding affinity for the classical target *TYMS* (−7.5 kcal/mol). Molecular dynamics simulations further confirmed the stable binding conformation of the protein-ligand complexes. This study provides mechanistic insights into how the *Eubacterium eligens* group influences atopic dermatitis through vitamin precursor-mediated systemic immune modulation and identifies *GALE* as a novel therapeutic target. The findings provide mechanistic insights into the gut-skin axis and support developing precision medicine approaches integrating microbiome interventions with targeted pharmacotherapy for AD management.

**IMPORTANCE:**

Genetic-level evidence of gut microbiota causality in atopic dermatitis: this study established a causal relationship between specific gut microbiota and the risk of atopic dermatitis at the genetic level, providing strong genetic evidence for the “gut-skin axis” theory. GALE is identified as a novel therapeutic target with redefined methotrexate mechanism: molecular docking study unexpectedly found that GALE binding affinity of MTX was significantly higher than that of its classical target TYMS, suggesting that GALE may be an important but previously unrecognized target of MTX in the treatment of AD. Multi-omics integration framework reveals increased keratinocyte heterogeneity: integrating single-cell RNA sequencing and computational pharmacology provided a cellular and molecular basis for understanding the characteristics of chronicity and recurrence of the disease.

## INTRODUCTION

Atopic dermatitis (AD) represents one of the most prevalent chronic inflammatory skin disorders worldwide, affecting approximately 15–20% of children and 1–3% of adults globally (1). Characterized by persistent skin barrier dysfunction, intense pruritus, and recurrent eczematous lesions, AD significantly impacts patients' quality of life and poses substantial healthcare burdens (2). While traditional research has predominantly focused on local skin pathology and immune dysregulation, emerging evidence suggests that AD pathogenesis extends beyond dermatological boundaries to encompass complex systemic interactions, particularly through the bidirectional communication network known as the gut-skin axis (3).

The gut-skin axis represents a sophisticated biological communication system wherein gut microbiota composition and metabolic activity directly influence skin homeostasis and inflammatory responses (4). This axis operates through multiple interconnected mechanisms, including immune system modulation, metabolite production, and barrier function regulation. Gut microbiota-derived metabolites, particularly short-chain fatty acids, bile acid derivatives, and amino acid fermentation products, can traverse systemic circulation to modulate skin immune responses and epidermal differentiation processes (5–8). Recent clinical observations have demonstrated that AD patients frequently exhibit altered gut microbiome diversity and composition compared to healthy individuals, suggesting that intestinal dysbiosis may contribute to disease initiation and perpetuation (9, 10). However, despite growing recognition of this gut-skin connection, the causal relationship between specific microbial taxa and AD risk remains largely unexplored, limiting the development of targeted microbiome-based therapeutic interventions.

Current therapeutic approaches for AD primarily rely on topical corticosteroids, systemic immunosuppressants, and emerging biologics targeting specific inflammatory pathways (11). While these treatments can provide symptomatic relief, they often fail to address underlying pathophysiological mechanisms and may produce significant adverse reactions during long-term use (12). Recent advances in understanding the molecular basis of AD have identified several key regulatory proteins that may represent novel therapeutic targets. UDP-galactose-4-epimerase (*GALE*) plays an essential role in galactose metabolism and has been implicated in both galactosemia and breast cancer through its effects on cellular metabolic processes (13). Similarly, the nuclear receptor *NR4A1* serves as an important regulator of inflammatory responses, particularly through its suppression of NF-κB signaling pathways in conditions, such as psoriasis and various cancers (14–17). Notably, in AD, decreased *NR4A1* expression correlates with persistent Th2-mediated inflammatory responses (18). The interconnected roles of these proteins, particularly *GALE*’s metabolic functions, highlight important mechanistic links between gut microbiota dysregulation and atopic dermatitis pathogenesis.

This study adopts an integrated multi-omics strategy combining gut microbiota with single-cell transcriptomics, reverse drug prediction, molecular docking, and molecular dynamics simulation to establish causal relationships between gut microbiota and AD, identify key bridging genes in the gut-skin axis, and discover potential therapeutic compounds targeting these critical pathways. Through a systematic investigation of microbial metabolite-gene interactions and their downstream effects on keratinocyte biology, this research aims to elucidate the molecular architecture of gut-skin communication and provide a foundation for developing next-generation therapies that target the root causes rather than merely the symptoms of atopic dermatitis.

## MATERIALS AND METHODS

### Data acquisition and integration

Single-cell transcriptomic data were sourced from GEO database GSE269981, including skin biopsy samples from five atopic dermatitis patients and four healthy controls, sequenced using the 10× Genomics platform (19).

### Identification and screening of key genes in the "gut-skin axis"

Metabolite information was systematically collected for significant gut microbiota taxa (*Sellimonas* genus and *Eubacterium rectale* group). *Sellimonas* metabolites included short-chain fatty acids, branched-chain fatty acids, organic acids, gaseous products, bile acid metabolites, and amino acid fermentation products. The *Eubacterium rectale* group metabolites encompassed butyrate, secondary bile acids, polyphenol metabolites, ferulic acid metabolites, and vitamin precursors.

Target genes and signaling pathways of these metabolites were identified using the STRING protein interaction database, KEGG pathway database, and bioinformatics tools. For STRING database analysis, protein-protein interactions were retrieved with the following criteria: interaction confidence score ≥ 0.4 (medium confidence threshold), restricted to *Homo sapiens* (taxonomy ID: 9606), and limited to interactions supported by experimental evidence, database annotations, or co-expression data. Text-mining-only predictions without experimental validation were excluded. For the KEGG pathway enrichment analysis, pathways were included if they met the following criteria: adjusted *P*-value < 0.05 after Benjamini-Hochberg multiple testing correction, contained at least three genes from the metabolite-related gene set, and were directly relevant to immune regulation, inflammatory responses, or skin barrier function. Pathways unrelated to atopic dermatitis pathogenesis were systematically excluded. Intersection analysis between metabolite-related genes and atopic dermatitis susceptibility genes identified key bridging genes associated with both gut microbiota metabolism and disease pathogenesis.

### Single-cell transcriptome data processing and cell type identification

Single-cell data were analyzed using the Seurat package (v4.3.0) (20). Quality control filtered cells with nFeature_RNA > 50 and percent.mt < 15. Data were normalized using ScaleData, principal components extracted via RunPCA, and batch effects corrected using RunHarmony. The top 20 principal components were used for FindNeighbors analysis and FindClusters function clustering at resolution 0.6. Cell type annotation was performed using the SingleR package with multiple reference data sets (Human_all, Hematopoietic, DatabaseImmuneCellExpression) to enhance accuracy (21).

### Intercellular communication network analysis

CellChat (v1.6.1) was used to analyze intercellular communication networks in atopic dermatitis skin. CellChat objects were created using integrated gene expression and cell type annotation data, including only secreted signaling molecules from the CellChatDB human ligand–receptor database (22). Overexpressed ligands and receptors were identified (thresholds: expression > 0.1, fold change > 0.25). Communication probabilities were calculated based on gene expression and inferred spatial proximity, with a minimum threshold of 10 cells per type for robust statistical analysis. Aggregate pathway-level communication strength was calculated by integrating multiple ligand-receptor pairs within each signaling pathway.

### Pseudotime trajectory analysis

To reconstruct the dynamics of cell state transitions during atopic dermatitis progression, pseudotime trajectory analysis was performed on preprocessed single-cell data. Trajectories were inferred based on UMAP dimensionality reduction, followed by minimum spanning tree construction using transcriptomic similarity. Cells were ordered based on dynamic gene expression patterns, with pseudotime values ranging from 0.0 (early/healthy state) to 10.0 (late/diseased state). Genes with significant expression changes at distinct pseudotime stages were identified, and functional enrichment analysis was conducted to reveal key biological processes involved in disease development.

### Functional enrichment analysis and reverse drug prediction

Systematic functional annotation analysis of identified key genes was performed using the clusterProfiler R package (23). Gene Ontology enrichment analysis encompassed three levels: biological processes, molecular functions, and cellular components. KEGG pathway enrichment analysis evaluated gene enrichment in metabolic pathways and signal transduction pathways. Enrichment analysis employed hypergeometric distribution testing, with *P*-values corrected for multiple testing using the Benjamini-Hochberg method and adjusted *P*-values < 0.05 set as the significance threshold. Based on the seven key genes identified through integrative analysis (*AKR1C2*, *GALE*, *GGH*, *NR4A1*, *P4HA1*, *PLA2G4B*, *TYMS*), systematic drug prediction was conducted by integrating the Comparative Toxicogenomics Database and the Drug-Gene Interaction Database. Candidate drugs potentially targeting these genes were identified through analysis of drug-gene interaction relationships. The drug screening strategy prioritized compounds capable of simultaneously modulating gut microecological balance and ameliorating skin inflammatory responses.

### Molecular docking and molecular dynamics simulation validation

#### Molecular docking analysis

Based on drug prediction results, methotrexate, fluorouracil, and tamoxifen were selected as candidate compounds for molecular docking studies. AutoDock Vina 1.5.6 software was employed to perform molecular docking calculations between candidate drugs and key target proteins (*GALE*, *TYMS*, *GGH*, *NR4A1*, *AKR1C2*) (24). Three-dimensional protein structures were retrieved from the Protein Data Bank and subjected to structural optimization and energy minimization. Drug small-molecule structures were drawn using ChemDraw and converted to molecular docking-compatible formats using Open Babel. Docking calculations employed genetic algorithms to search for optimal binding conformations and evaluate drug-protein binding affinity. Complexes with binding energies below −5.0 kcal/mol were selected for further molecular dynamics simulation validation.

#### Molecular dynamics simulation

Complexes were subjected to 100 ns MD simulations using Gromacs 2022 (25). Proteins were parameterized using the CHARMM 36 force field, while ligand topologies were constructed using GAFF2 force field parameters. Periodic boundary conditions were applied, with protein-ligand complexes placed in cubic boxes. The TIP3P water model was used to solvate the system, creating water boxes with periodic boundaries of 1.2 nm (26). Electrostatic interactions were treated using the particle mesh Ewald (PME) method and Verlet algorithms. Subsequently, 100,000 steps of NVT ensemble equilibration and NPT ensemble equilibration were performed with coupling constants of 0.1 ps for 100 ps simulations. van der Waals and Coulombic interactions were calculated using cutoff values of 1.0 nm. Finally, the system underwent molecular dynamics simulation at constant temperature (310 K) and pressure (1 bar) for a total duration of 100 ns. Binding stability of complexes was evaluated by analyzing parameters, including RMSD, RMSF, hydrogen bond number, radius of gyration, and solvent-accessible surface area.

### Quantitative PCR validation

Atopic dermatitis cell models were established and divided into control (K), model (M), and methotrexate-treated (Y) groups. Cells were washed with PBS and lysed with RNA extraction solution, and total RNA was extracted using the chloroform-isopropanol precipitation method. RNA concentration was determined by NanoDrop 2000 and diluted to 200 ng/µL (detailed protocols in Table S1).

Reverse transcription was performed using SweScript All-in-One RT SuperMix (Wuhan Saisel, G3337) in 20 µL reaction system at 42°C for 30 min. Quantitative PCR was conducted using SYBR Green Master Mix (Wuhan Saisel, G3326) on a Bio-Rad CFX Connect system with initial denaturation at 95°C for 30 s, followed by 40 cycles of 95°C for 15 s and 60°C for 30 s. Each sample was analyzed in triplicate, and gene expression was calculated using 2^(−ΔΔCT) method with actin as reference gene.

## RESULTS

### Quality control and analysis of single-cell RNA sequencing data

**Fig 1:**
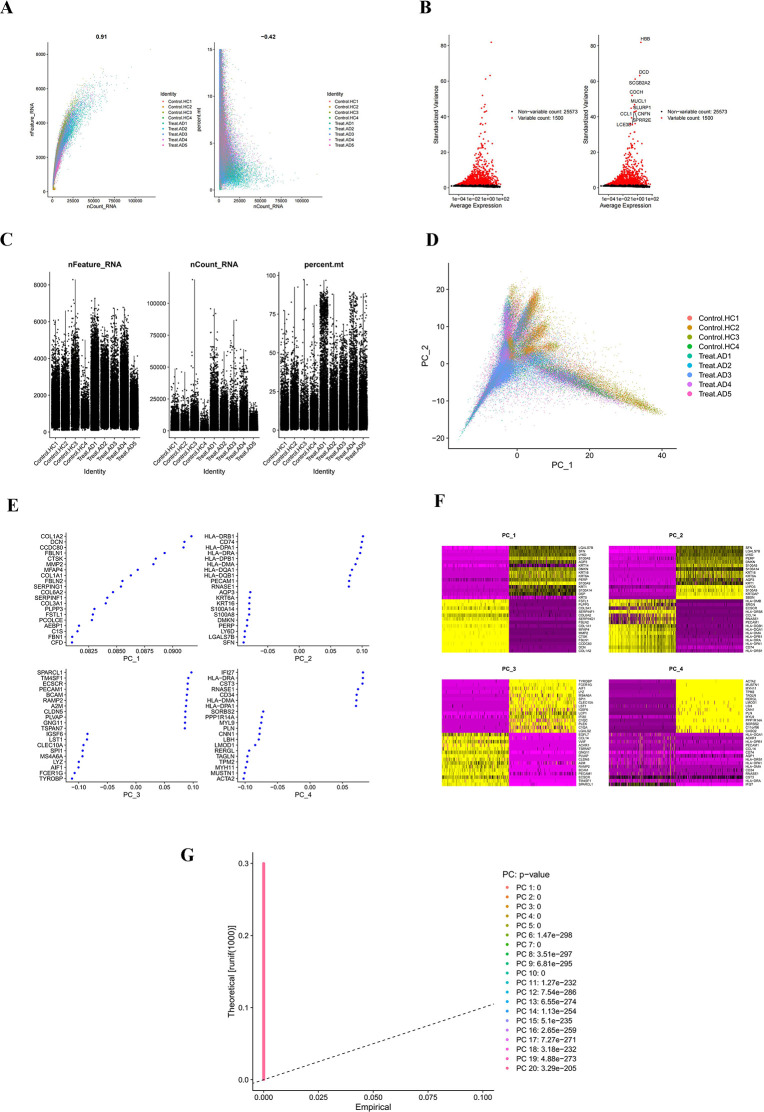
Quality control and principal component analysis of single-cell RNA: (**A**) scatter plots showing the correlation between the total RNA count per cell and the number of genes detected. Different colors represent the control group (Control.HC1–HC4) and the atopic dermatitis group (Treat.AD1–AD5). (**B**) Hypervariable gene identification map. (**C**) Violin plot showing the distribution of three main quality control indicators (nFeature RNA, nCount RNA, and percent.mt) among samples. (**D**) Principal component analysis (PCA) plots showing the distribution of cells on the first two principal components. (**E–G**)Genetic scatter plot and heatmap. Colors indicate different sample sources.

**Fig 2:**
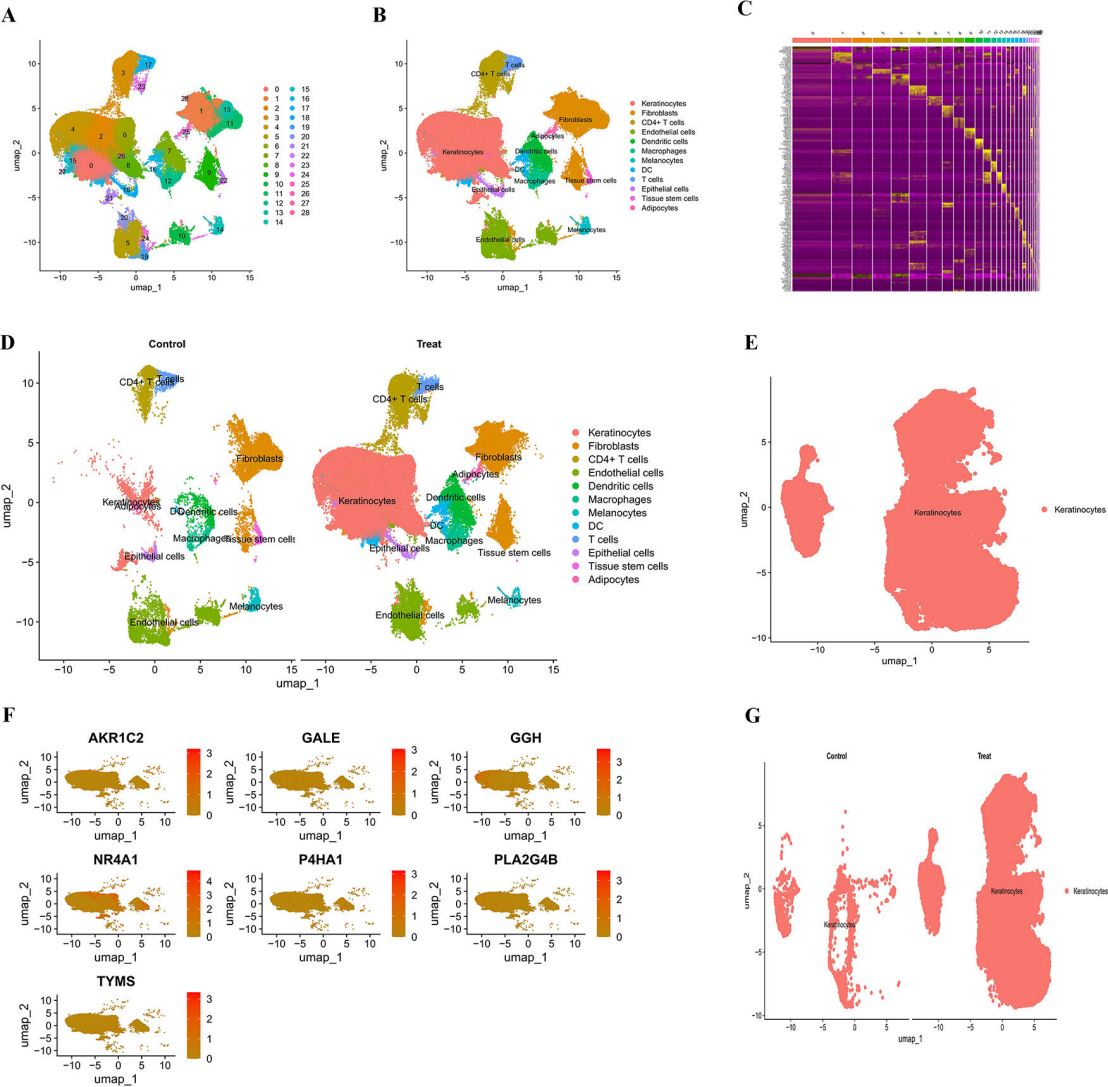
Cell clustering, annotation, and disease comparative analysis: (**A**) UMAP-based cell clustering results, with each dot representing one cell and different colors representing 29 different cell clusters (0–28); (**B**) annotation results of cell types showed the main cell types; (**C**) heat map of differentially expressed genes in each cell cluster; (**D**) UMAP analysis of group comparisons, with the control group on the left and the atopic dermatitis group on the right; (**E**) specific distribution map of keratinocytes; (**F**) expression distribution of seven key genes (*AKR1C2*, *GALE*, *GGH*, *NR4A1*, *P4HA1*, *PLA2G4B*, and *TYMS*) in UMAP space; and (**G**) comparison of distributions of keratinocytes between control and atopic dermatitis groups (UMAP, uniform manifold approximation and projection; AKR1C2, Aldo-Keto Reductase Family 1 Member C2; GALE, UDP-galactose-4′-epimerase; GGH, gamma-glutamyl hydrolase; NR4A1, Nuclear Receptor Subfamily 4 Group A Member 1; P4HA1, Prolyl 4-Hydroxylase Alpha Subunit 1; PLA2G4B, Phospholipase A2 Group IVB; TYMS, thymidylate synthase).

Stringent quality control and feature analysis were performed on the single-cell RNA sequencing data, as illustrated in Fig. 1. The scatter plot on the left of Fig. 2A shows a strong positive correlation (correlation coefficient 0.91) between total RNA counts (nCount_RNA) and the number of detected genes (nFeature_RNA) per cell, indicating stable sequencing quality and sufficient depth. Different colors represent control (Control. HC1–HC4) and atopic dermatitis (Treat. AD1–AD5) groups, all showing similar distributions and demonstrating effective batch effect control.

As shown in Fig. 1B, analysis of gene expression variability identified a subset of highly variable genes (highlighted in red), such as HBB and DCD, which may play key roles in cell heterogeneity, while most genes showed a relatively stable expression. Violin plots in Fig. 1C display the distributions of three major quality control metrics (nFeature_RNA, nCount_RNA, and percent.mt) across samples. Most cells fell within reasonable ranges, especially for percent.mt, confirming overall data reliability and high cell viability. Based on these results, we set filtration thresholds of nFeature_RNA > 50 and percent.mt < 15 to remove low-quality cells. Figure 1D shows the distribution of cells along the first two principal components after PCA, with colors indicating different sample origins. The observed mixing of sample groups suggests that clustering is governed predominantly by biological variation, rather than technical batches. Based on these quality control and preprocessing results, we confirmed that the single-cell RNA sequencing data were of high quality and suitable for subsequent analyses.

### Single-cell UMAP dimensionality reduction and cell type annotation

Based on integrated principal component analysis and batch correction, we performed clustering and cell type annotation on the single-cell RNA-seq data, as shown in Fig. 2. Figure 2A displays the UMAP-based clustering results, where each dot represents an individual cell, and the different colors indicate distinct clusters; a total of 29 clusters (0–28) were identified. These clusters show a clear separation in the UMAP space, suggesting that they represent different cell types or cellular states.

Cell type annotation was performed for each cluster using marker gene expression profiles and SingleR automatic annotation results (Fig. 2B). Major annotated cell types included keratinocytes (representing the largest population), fibroblasts, CD4+ T cells, endothelial cells, dendritic cells, macrophages, melanocytes, DC cells, T cells, epithelial cells, tissue stem cells, and adipocytes, covering the major cell types present in the skin tissue. Figure 2C shows a heatmap of differentially expressed genes across clusters.

To compare the cellular compositions between atopic dermatitis patients and healthy controls, group-stratified UMAP analysis was conducted (Fig. 2D). The left panel shows cell distribution in controls, and the right panel shows that in the atopic dermatitis group. Notably, the cell composition differed between groups: keratinocytes appeared more dispersed in the atopic dermatitis group, suggesting increased cellular heterogeneity associated with disease. In addition, CD4+ T and dendritic cells were more abundant in the atopic dermatitis samples, consistent with the immunopathology of the disease. Differences were also observed for endothelial and fibroblast cell populations, indicating vascular and stromal remodeling in disease conditions. Figure 2E specifically focuses on the distribution of keratinocytes, which are the primary contributors to skin barrier function and play a key role in atopic dermatitis pathogenesis. Figure 2F presents the UMAP spatial expression of seven key genes (*AKR1C2*, *GALE*, *GGH*, *NR4A1*, *P4HA1*, *PLA2G4B*, *TYMS*), with the color intensity indicating the expression level from low (yellow) to high (red). These genes showed variable expression patterns in keratinocytes, suggesting their involvement in the disease process.

Finally, we compared keratinocyte distributions between control and atopic dermatitis groups (Fig. 2G). Keratinocytes in the atopic dermatitis group displayed greater heterogeneity and a more scattered distribution, which may reflect changes in cellular function and differentiation status characteristic of disease and is likely related to skin barrier dysfunction—a hallmark feature of atopic dermatitis.

### Cell-cell communication and identification of key “gut–skin axis” genes

**Fig 3:**
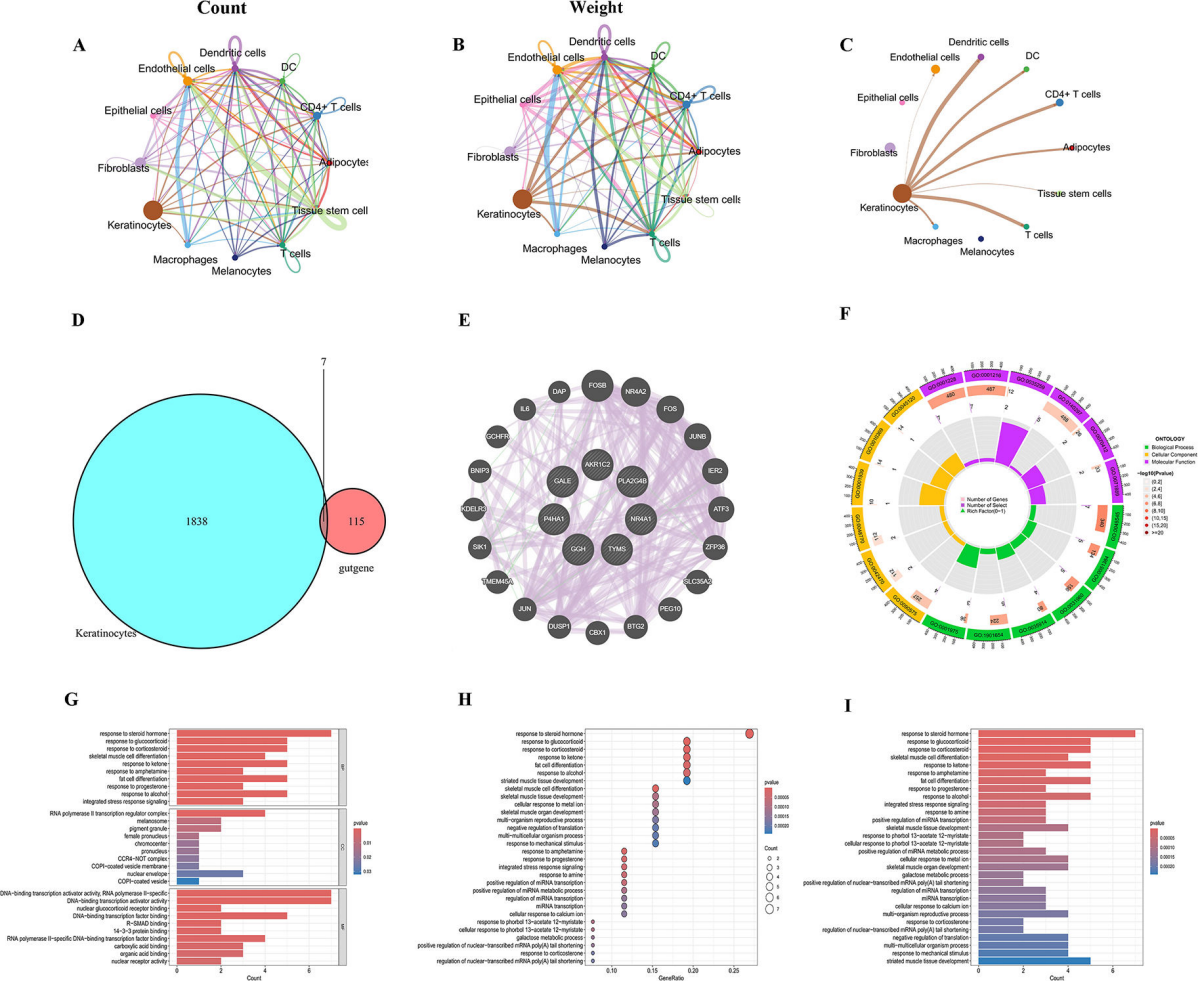
Intercellular communication analysis and gene function network analysis: (**A**) diagram of cell communication network based on cell count, with node size indicating cell number; (**B**) diagram of cell communication network weighted based on interaction strength; (**C**) diagram of communication connections between keratinocytes and other cell types; (**D**) Venn diagram showing intersection analysis of 1,838 keratinocyte differentially expressed genes, and 115 gut microbiome-related genes identified seven key bridging genes; (**E**) regulatory network diagram of key gene interaction, including *AKR1C2*, *GALE*, *GGH*, *NR4A1*, *P4HA1*, *PLA2G4B*, *TYMS*, and their related regulators; (**F**) ring diagram of key genes functional enrichment; (**G**) GO biological process enrichment analysis; (**H**) GO functional enrichment analysis; and (**I**) KEGG pathway enrichment analysis (GO, Gene Ontology; KEGG, Kyoto Encyclopedia of Genes and Genomes).

**Fig 4:**
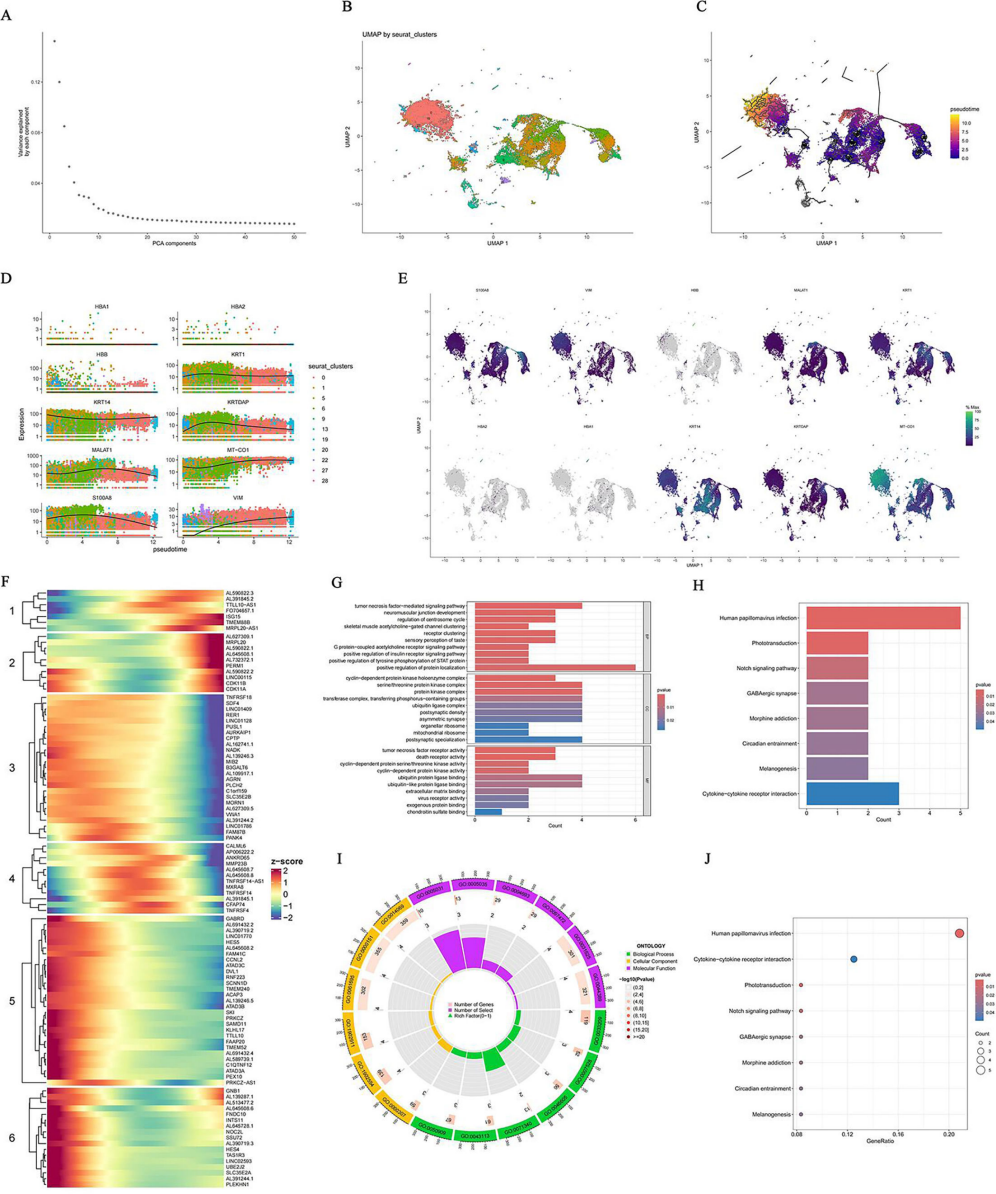
Pseudotime trajectory analysis: (**A**) variance contribution plot of the first 20 principal components; (**B**) cell cluster distribution map based on UMAP; (**C**) result of pseudo-time trajectory inference quantifying the relative position of each cell in disease progression; (**D**) expression trend plot of major marker genes along the proposed time trajectory; (**E**) expression distribution of seven key genes (*AKR1C2*, *GALE*, *GGH*, *NR4A1*, *P4HA1*, *PLA2G4B*, and *TYMS*) in the proposed time trajectory; (**F**) clustering of dynamic gene expression heatmaps identified six gene clusters (markers 1–6), each with a distinct expression pattern; (**G**) GO biological process enrichment analysis; (**H**) KEGG enrichment analysis; and (**I, J**) results of supplemental functional enrichment analysis.

Cellular communication network analysis revealed complex intercellular interaction patterns in atopic dermatitis, as shown in Fig. 3A through C. Figure 4A presents the communication network based on cell counts, Fig. 3B is weighted by interaction strength, and Fig. 3C highlights the connections between keratinocytes and other cell types. Each node represents a cell type, including keratinocytes, fibroblasts, endothelial cells, dendritic cells, DC cells, CD4+ T cells, adipocytes, tissue stem cells, macrophages, and melanocytes. The analysis showed that keratinocytes are central within the network, directly connecting with almost all other cell types, underscoring their coordinating role in the skin microenvironment. Fibroblasts and endothelial cells also display broad interactions, while immune cells, such as CD4+ T cells, dendritic cells, and macrophages, form a closely associated subnetwork, likely reflecting immune collaboration. Compared with controls, the atopic dermatitis group showed more prominent immune cell involvement and more complex communication, consistent with increased immune activation in disease.

To identify molecular bridges between the gut microbiota and skin pathology, we performed an intersection analysis between differentially expressed genes in keratinocytes and gut microbiota-associated metabolic genes. As shown in Fig. 3D, comparison of 1,838 keratinocyte DEGs and 115 gut microbiota-related genes ("gutgenes") identified seven key genes (*AKR1C2*, *GALE*, *GGH*, *NR4A1*, *P4HA1*, *PLA2G4B*, *TYMS*), which are both linked to gut microbial metabolites and differentially expressed in AD keratinocytes. These genes form an interacting regulatory network (Fig. 3E) that also includes partners, such as *DAP*, *FOSB*, *NR4A2*, *FOS*, *JUN*, and *ATF3*—most of which are involved in transcriptional regulation and inflammation, suggesting they may cooperate in AD pathogenesis. Functional enrichment of these key genes depicted in the circular diagram in Fig. 3F indicates their involvement in biological processes like transcriptional regulation, cell cycle, and inflammatory responses.

GO enrichment analyses (Fig. 3G through I) highlight significant enrichment in transcription regulation (particularly RNA polymerase II-related), cell cycle control, nucleotide metabolism, and cell proliferation. Molecular functions include DNA-binding transcription activator activity, CCAAT/enhancer-binding protein activity, and cytokine response element binding. Notably, pathways relevant to cytokine signaling and inflammation were prominent, linking these genes to immune modulation and the inflammatory milieu common in AD. KEGG pathway analyses (Fig. 3I) show enrichment in cell cycle, apoptosis, PPAR signaling, and several inflammation-related pathways, including IL-17 and TNF signaling, further supporting their role in disease development.

### Pseudotime analysis of keratinocytes and dynamic gene expression changes

Pseudotime analysis revealed clear patterns of cell state transitions in atopic dermatitis, as illustrated in Fig. 4. Figure 4A displays the variance contribution of the top 20 principal components, guiding dimension selection for trajectory construction. Figure 4B shows a UMAP-based distribution of cell clusters, each represented by a distinct color. Trajectory inference results (Fig. 4C) visualize pseudotime progression, with color gradients from dark purple (early state, pseudotime = 0.0) to bright yellow (late state, pseudotime = 10.0), quantifying each cell’s relative position during disease progression. These trajectories demonstrate ordered transitions in cell states rather than random inflammatory activation.

Distinct developmental or disease progression trajectories can be observed, where early pseudotime states (dark purple) are mainly central and right on UMAP, likely representing baseline or steady-state cells. Intermediate states (pink-red, values 2.5–7.5) bridge clusters and may correspond to cells undergoing active transitions, while late pseudotime states (bright yellow, near 10.0) are localized to the upper left, potentially representing terminal differentiation or highly activated states in atopic dermatitis. Multiple trajectory branches indicate diverse cell fate decisions or reactive pathways. The central area contains several branching points, suggesting alternative cellular responses to inflammation. Gray lines connecting clusters represent inferred paths between cell states, underscoring the continuity and complexity of transitions during disease. Figure 4D presents expression trends of major marker genes along the pseudotime trajectory, with different colors for different clusters. Some genes exhibit expression changes at early stages, while others rise or fall notably at later stages, providing insights into regulatory network dynamics during disease progression.

Figure 4E illustrates expression distributions of the seven key genes (*AKR1C2*, *GALE*, *GGH*, *NR4A1*, *P4HA1*, *PLA2G4B*, *TYMS*) across pseudotime. Certain genes, such as *GALE* and *P4HA1*, change early, while others like *NR4A1* and *GGH* upregulate mainly during later stages—suggesting that early-changing genes may contribute to AD initiation, whereas late-changing genes may be involved in disease progression or maintenance. The heatmap in Fig. 4F shows clustered gene expression dynamics across pseudotime, identifying six gene clusters (labeled 1−6), each with distinct expression patterns—some peak early (clusters 1 and 2), others in the intermediate phase (clusters 3 and 4), and some only at late stages (clusters 5 and 6). These patterns shed light on the temporal regulation of gene networks in the evolution of AD.

GO and KEGG enrichment analyses (Fig. 4G through J) revealed that pseudotime-related genes are enriched in various functional pathways. GO biological process analysis (Fig. 4G) shows involvement in processes, such as motor neuron axon-mediated signaling, neuromuscular processes, and skeletal muscle contraction. KEGG enrichment (Fig. 4H) highlights significant pathways, including HPV infection, phototransduction, Notch signaling, GABAergic synapse, and morphine addiction.

### Reverse drug prediction analysis

**Fig 5:**
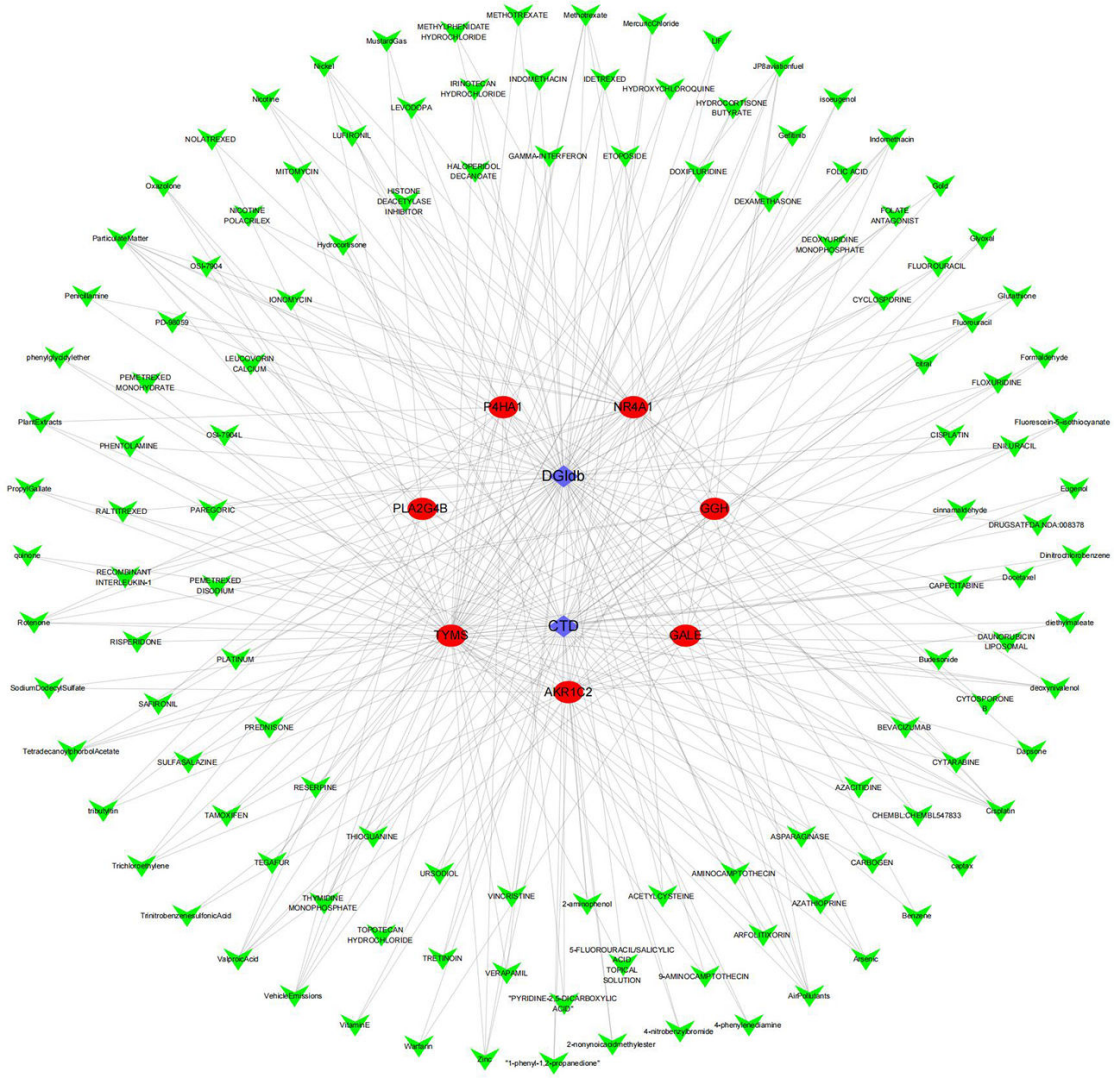
Reverse drug prediction network diagram. The drug-gene interaction network diagram constructed using Cytoscape (v3.9.1) contains 72 nodes (seven genes and 65 drugs) and 148 edges.

Significant candidate drugs were identified from two databases, including methotrexate (targeting *TYMS*, *GALE*, and *GGH*), fluorouracil (targeting *TYMS* and *GGH*), and tamoxifen (targeting *NR4A1* and *AKR1C2*). In addition, drugs, such as hydrocortisone, cisplatin, and dexamethasone, were also predicted to interact with multiple genes. The results of the drug-gene interaction network analysis can be seen in Fig. 5.

A drug-gene interaction network was constructed using Cytoscape (v3.9.1) to visualize the associations between key genes and candidate drugs (27). The network comprised 72 nodes (seven genes and 65 drugs) and 148 edges. Methotrexate, fluorouracil, and tamoxifen showed high connectivity (degree > 3), indicating interactions with several target genes. Further topological analysis using the "Network Analyzer" tool revealed that these three drugs occupied central positions in the regulatory network, as indicated by high betweenness and closeness centrality values.

### Molecular docking results

**Fig 6:**
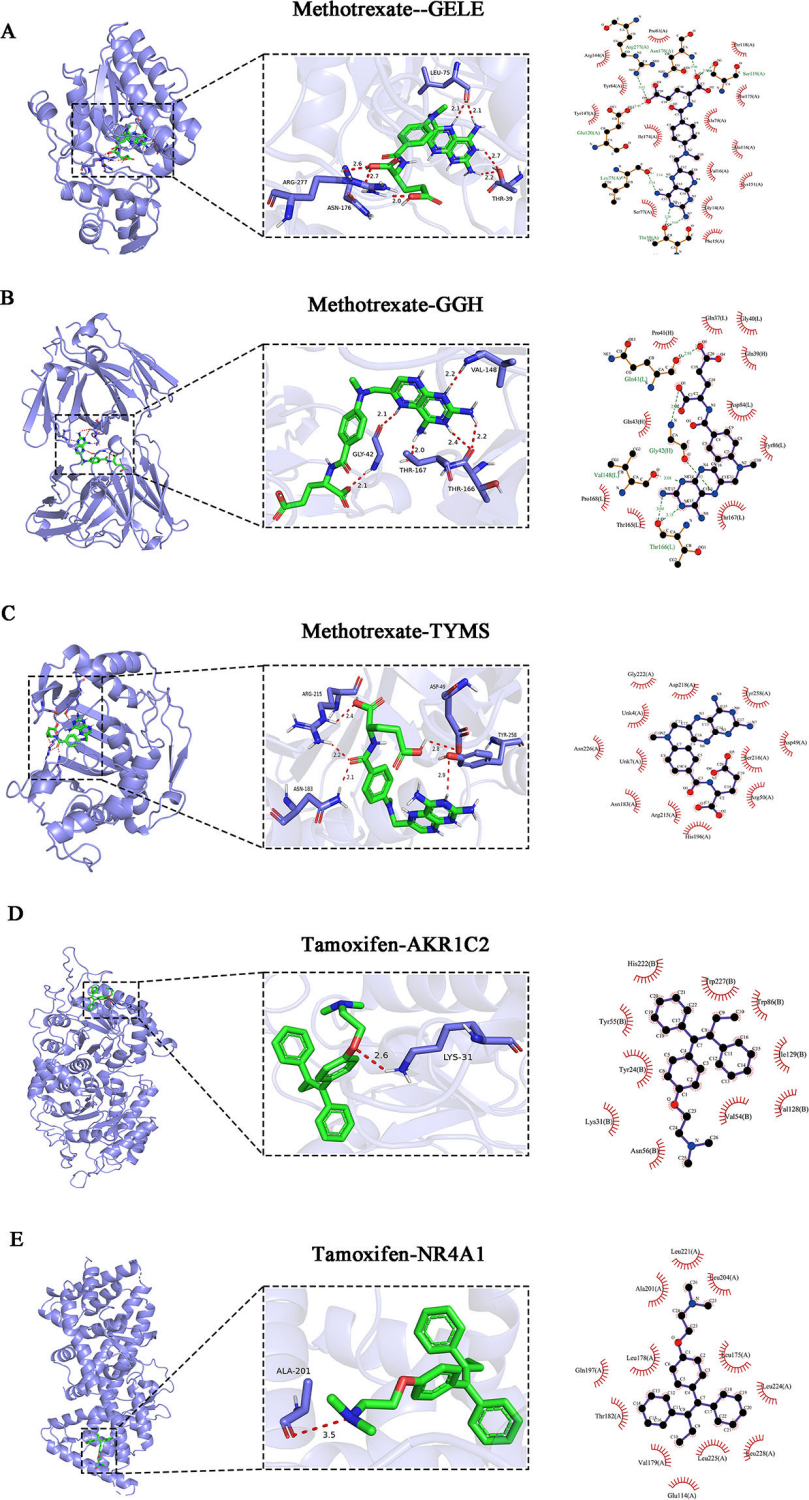
Core target and drug molecule docking results: (**A**) Methotrexate-gale complex (binding energy: –10.4 kcal/mol), (**B**) methotrexate-TYMS complex (binding energy: –7.5 kcal/mol), (**C**) methotrexate-GGH complex (binding energy: –7.7 kcal/mol), (**D**) Tamoxifen-akr1c2 complex (binding energy: –7.4kcal/mol), (**E**) tamoxifen-NR4A1 complex (binding energy: –7.2 kcal/mol).tamoxifen-*NR4A1* complex (binding energy: –7.2 kcal/mol).

Molecular docking was employed to explore the optimal binding modes between candidate drugs and target proteins relevant to atopic dermatitis. As shown in Fig. 6, methotrexate was docked with *GALE*, *TYMS*, and *GGH*, while tamoxifen was docked with *NR4A1* and *AKR1C2* using AutoDock 1.5.6. All five simulations yielded low binding energies (affinity < −5 kcal/mol), indicating strong interactions. The methotrexate-GALE complex exhibited the strongest binding affinity (−10.4 kcal/mol), followed by methotrexate-GGH (−7.7 kcal/mol), methotrexate-TYMS (−7.5 kcal/mol), tamoxifen-AKR1C2 (−7.4 kcal/mol), and tamoxifen-NR4A1 (−7.2 kcal/mol). These results suggest potent interactions between both drugs and their respective protein targets, implying that methotrexate and tamoxifen may influence atopic dermatitis mechanisms via these interactions.

Structural visualization using PyMOL further confirmed close contact and specific binding conformations. Detailed 2D interaction analysis showed that the drug molecules formed stable hydrogen bonds (2.0–3.5 Å) with key amino acid residues, such as ARG-277, ASN-176, THR-39, LEU-75, GLY-42, THR-166, THR-167, VAL-148, ARG-215, ASN-183, TYR-258, ASP-49, LYS-31, and ALA-201. In addition, significant van der Waals interactions with surrounding hydrophobic residues (e.g., Leu, Val, Tyr, Trp, Ile, Pro) further stabilized the drug-protein complexes. Aromatic residues (Tyr, Trp, Phe) contributed hydrophobic and potential π-π stacking interactions, enhancing specificity and binding stability. Collectively, these specific interactions underpin the strong affinity observed, supporting the potential of methotrexate and tamoxifen to modulate key protein targets linked to atopic dermatitis and gut microbiota-related mechanisms.

### Molecular dynamics simulation results

The four complex groups of methotrexate-*GALE*, methotrexate-*TYMS*, methotrexate-*GGH,* and tamoxifen-*NR4A1* were selected for *molecular* dynamics modeling in this study based on the following considerations: molecular docking results showed that the binding energy of *GALE* with MTX (−10.4 kcal/mol) was significantly higher than that of its traditional classical target *TYMS* (−7.5 kcal/mol), suggesting that *GALE* may be an important but not fully understood target of MTX in the treatment of AD. Considering the role of *GALE* in glucose metabolism and its potential association with skin barrier function, this finding may provide a new explanation for the therapeutic mechanism of methotrexate. The binding energy with *GGH* (−7.7 kcal/mol) also met the threshold criteria for drug-target interaction. For tamoxifen, its combination with *NR4A1* was chosen for simulation (binding energy of −6.5 kcal/mol) primarily based on the critical regulatory role of *NR4A1* in Th2-type immune responses, which are highly relevant to the pathogenesis of atopic dermatitis. In contrast, the binding of tamoxifen to *AKR1C2*, although stable (binding energy − 5.8 kcal/mol), was weakly associated with disease and was, therefore, not included in the kinetic simulation analysis.

**Fig 7:**
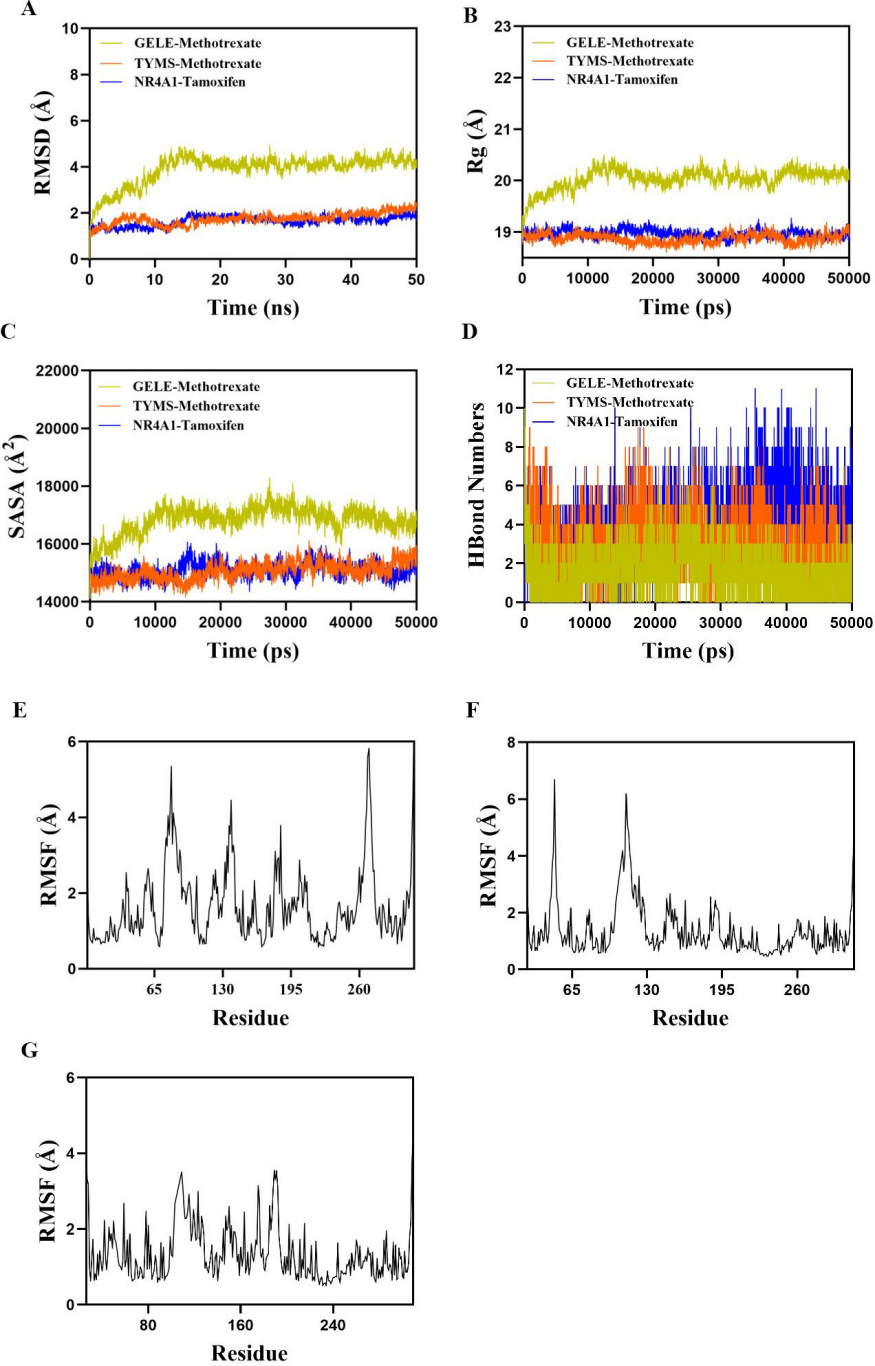
Molecular dynamics simulation of protein-ligand complexes. (**A**) RMSD values of protein-ligand complexes over time; (**B**) Rg values of protein-ligand complexes over time; (**C**) SASA values of protein-ligand complexes over time; (**D**) HBonds values of protein-ligand complexes over time; and (**E**) RMSF values of amino acid backbone atoms in the GELE-Methotrexate complex; (**F**) RMSF values of amino acid backbone atoms in the TYMS-Methotrexate complex; (**G**) RMSF values of amino acid backbone atoms in the NR4A1-Tamoxifen complex (RMSD, root mean square deviation; Rg, radius of gyration; SASA, solvent accessible surface area; HBonds, hydrogen bonds; RMSF, root mean square fluctuation).

Root mean square deviation (RMSD) was used to evaluate conformational stability of protein-ligand systems. As shown in Fig. 7A, the *GALE*-methotrexate, *TYMS*-methotrexate, and *NR4A1*-tamoxifen complexes all reached equilibrium within 20 ns, with RMSD fluctuations stabilizing at approximately 4, 2.5, and 2 Å, respectively, indicating overall structural stability. Analyses of radius of gyration (Rg) and solvent accessible surface area (SASA) (Fig. 7B and C) revealed only minor fluctuations during the simulation period, with no significant expansion or contraction of the complexes.

Hydrogen bonding plays an important role in the binding of ligands to proteins. Figure 7D shows the number of hydrogen bonds formed during simulation: the *GALE*-methotrexate complex typically maintained ~4 hydrogen bonds (range 0–7), the TYMS-methotrexate complex ~6 bonds (range 0–9), and the *NR4A1*-tamoxifen complex up to ~8 bonds (range 0–11), supporting strong protein–ligand interactions. Root mean square fluctuation (RMSF) analysis (Fig. 7E through G) indicated generally low flexibility of amino acid residues for all three protein–ligand complexes (mostly below 4 Å), further supporting high conformational stability.

In summary, *GALE*-methotrexate, *TYMS*-methotrexate, and *NR4A1*-tamoxifen complexes demonstrated stable binding and favorable hydrogen bonding patterns, suggesting that methotrexate and tamoxifen interact robustly with *GALE*, *TYMS*, and *NR4A1* and are potentially effective modulators in the context of atopic dermatitis.

### Experimental validation of *GALE* expression

To validate our computational findings regarding *GALE* as a potential therapeutic target, we performed a quantitative PCR analysis using an atopic dermatitis cell model. As shown in Table S2, *GALE* expression levels were measured across three experimental conditions: control group (K), atopic dermatitis model group (M), and methotrexate-treated group (Y).

#### Cell source and model construction

We used the human keratinocyte cell line HaCaT cells (purchased from the Cell Bank of the Chinese Academy of Sciences), which is widely used in *in vitro* studies of atopic dermatitis. The atopic dermatitis cell model was established by co-stimulating with TNF-α (10 ng/mL) and IL-4 for 48 h. This method can effectively simulate the inflammatory microenvironment of atopic dermatitis and the pathological state of keratinocytes.

#### Experimental grouping and sample size

The experiment was set up with three groups, each with six biological replicates (*n* = 6): the control group (K) was normal cultured HaCaT cells; the model group (M) was AD model cells established by TNF-α and IL-4 stimulation; and the treatment group (Y) was AD model cells treated with methotrexate (50 µM, for 24 h). Each sample was subjected to three technical replicates to ensure the reliability of the detection results.

#### Detailed experimental procedure

RNA extraction was performed using the chloroform-isoamyl alcohol precipitation method; reverse transcription was carried out using SweScript All-in-One RT SuperMix (G3337, Wuhan Saver); and qPCR was conducted using SYBR Green Master Mix (G3326, Wuhan Saver) on the Bio-Rad CFX Connect system. Gene expression was calculated using the 2^(−ΔΔCT) method, with ACTB as the internal reference gene.

The results revealed that *GALE* expression in the atopic dermatitis model group showed only a modest increase compared to controls (fold change = 1.16, representing a 16% increase), suggesting that *GALE* dysregulation may not be a primary driver in AD pathogenesis. However, methotrexate treatment dramatically upregulated *GALE* expression by 4.04-fold compared to controls (304% increase), providing direct experimental evidence supporting our molecular docking predictions that methotrexate exerts therapeutic effects through *GALE* pathway activation. These findings indicate that while *GALE* may not be significantly altered in the disease state itself, methotrexate’s therapeutic mechanism in atopic dermatitis may involve a substantial enhancement of *GALE* enzymatic activity, potentially restoring normal galactose metabolism and improving skin barrier function through metabolic rebalancing rather than correcting an existing deficiency.

## DISCUSSION

This study employed a multi-omics integration strategy to establish, for the first time at the genetic level, a causal relationship between gut microbiota and atopic dermatitis, yielding findings of significant theoretical and clinical importance. The positive association between the *Eubacterium eligens* group and the *Sellimonas* genus with atopic dermatitis risk illuminates the central role of the gut-skin axis in disease pathogenesis. Notably, these two microorganisms are typically considered beneficial in healthy populations, yet may transform into pathogenic factors under specific genetic backgrounds or environmental conditions. This aligns with Fang, who found that *Bifidobacterium longum* modulates AD via tryptophan metabolism, yet our study uniquely establishes causality at the genetic level, advancing beyond observational associations (8). The *Eubacterium eligens* group primarily produces short-chain fatty acids, such as butyrate, which normally exert anti-inflammatory effects (28). However, excessive proliferation may lead to significant reduction in intestinal pH, disrupting gut microecological balance and subsequently affecting systemic immune status through leaky gut syndrome. The *Sellimonas* genus mainly participates in protein fermentation, producing bioactive amines, including histamine and tryptamine (29). These metabolites can directly activate mast cells and eosinophils, promoting Th2-type immune responses (30, 31). This "double-edged sword" effect underscores the need for more precise and individualized approaches when developing gut microbiota-based therapeutic strategies.

Our research has established a causal relationship at the genetic level between the gut microbiota and atopic dermatitis, highlighting the central role of the gut-skin axis in the pathogenesis of the disease. The positive correlation between the *Eubacterium eligens* group and the *Sellimonas* genus and the risk of AD is consistent with the extensive literature on the dysbiosis of the gut microbiota in AD. Previous studies have shown that the composition of the gut microbiota in AD patients changes, with an increase in the proportion of *Clostridia* (including the *Eubacterium eligens* group) and a decrease in the proportion of *Bifidobacteria* (32). Moreover, *Sellimonas* participates in protein fermentation and produces bioactive amines, such as histamine, which can exacerbate the Th2-type immune response (33). Therefore, our findings provide further evidence for the crucial role of the gut-skin axis in the development of AD.

The increased keratinocyte heterogeneity revealed by single-cell analysis warrants deeper exploration of its biological significance. Traditional perspectives view keratinocytes primarily as physical barrier components (34), but our research demonstrates their more complex immunoregulatory role in atopic dermatitis. CellChat analysis revealed extensive ligand-receptor interactions between keratinocytes and immune cells, particularly the communication networks formed with CD4+ T cells, dendritic cells, and macrophages, which are significantly enhanced in disease states. This altered interaction pattern may represent a key mechanism in the transition from acute inflammation to chronicity. This is consistent with recent single-cell studies on AD, which confirmed that Th2/Th22 T cells are key drivers of AD inflammation (35). For instance, a single-cell RNA sequencing study comparing AD and psoriasis showed that AD was dominated by Th2/Th22 T cell populations, while psoriasis was dominated by Th17/Tc17 (36). Our data further revealed the specific communication network between keratinocytes and immune cells, suggesting that these interactions may drive the chronic inflammatory state in AD. Pseudotime trajectory analysis further revealed the continuous progression from early homeostatic to late activated states, where early-change genes, such as *GALE* and *P4HA1*, may participate in disease initiation, while late-upregulated *NR4A1* and *GGH* are more involved in inflammation maintenance and tissue remodeling. This temporal gene expression pattern suggests that atopic dermatitis is not merely an inflammatory disease but a complex pathological process involving disrupted epidermal differentiation programs and abnormal repair mechanisms. This understanding provides crucial insights into the chronic and recurrent characteristics of the disease and establishes a theoretical foundation for developing precision therapeutic strategies targeting different disease stages.

*GALE* emerges as a novel therapeutic target with considerable clinical translation potential deserving focused attention and in-depth investigation. Current research on *GALE*’s role in skin biology remains limited, primarily concentrated in congenital galactosemia studies (37). However, our findings suggest its involvement in atopic dermatitis pathogenesis may be far more significant than previously anticipated. *GALE*’s central position in the glycometabolic network establishes it as a critical molecular bridge connecting systemic metabolic status with local skin barrier function. From a mechanistic perspective, *GALE* dysfunction may affect skin homeostasis through multiple levels. First, abnormal UDP-galactose accumulation directly impacts glycosaminoglycan and proteoglycan synthesis, which are core components constituting the extracellular matrix and maintaining skin moisturizing function (38–41). Second, as a key substrate for glycoprotein glycosylation modifications, UDP-galactose metabolic disruption may lead to functional abnormalities in epidermal differentiation marker proteins, such as filaggrin and keratin, thereby compromising stratum corneum integrity. *GALE* dysfunction may disrupt skin barrier function by damaging glycosaminoglycan synthesis and epidermal differentiation, which is consistent with research on glycosylation in skin homeostasis (42). Third, *GALE* may also influence the proliferation-differentiation balance of keratinocytes by regulating cellular energy metabolic states, which is essential for maintaining normal epidermal renewal cycles.

Our experimental validation provides direct evidence supporting the computational predictions regarding methotrexate’s interaction with *GALE*. Quantitative PCR analysis revealed that while *GALE* expression showed only modest changes in the atopic dermatitis model (1.16-fold increase compared to controls), methotrexate treatment dramatically upregulated *GALE* expression by 4.04-fold. This finding suggests that *GALE*’s therapeutic relevance lies not in correcting disease-associated dysregulation, but rather in its capacity for metabolic enhancement following pharmacological intervention. The high-affinity binding between methotrexate and *GALE* substantially exceeds its binding strength with the classical target *TYMS*, providing a novel theoretical framework for explaining methotrexate’s unique efficacy in atopic dermatitis treatment. This finding extends Zhao et al., who described methotrexate’s immunosuppressive effects via dihydrofolate reductase inhibition, by proposing a dual mechanism involving skin barrier repair through *GALE* regulation (43). Traditional perspectives attribute methotrexate’s effects primarily to immunosuppression through dihydrofolate reductase inhibition (43). Our research suggests it may simultaneously improve skin barrier function directly through *GALE* activity regulation, creating synergistic therapeutic effects combining immunosuppression with barrier repair. This dual mechanism may explain why methotrexate’s therapeutic efficacy in atopic dermatitis often demonstrates longer duration compared to other immunosuppressants. From a drug development perspective, *GALE* as an enzyme target provides clear structural foundations and mechanisms for designing novel small-molecule drugs, particularly addressing the precision treatment needs for skin barrier dysfunction in atopic dermatitis patients. *GALE*-based drug design strategies may include enzyme activity modulators, allosteric regulators, or metabolic pathway redirectors, potentially developing next-generation atopic dermatitis therapeutics with reduced side effects and enhanced targeting specificity. Furthermore, as a key node in the gut-skin axis, *GALE* functional regulation may simultaneously improve both intestinal and skin barriers, achieving dual therapeutic benefits.

Also, the discovery of tamoxifen’s interaction with *NR4A1* provides new strategic options for atopic dermatitis treatment. *NR4A1*, as a ligand-independent nuclear receptor, plays important roles in regulating inflammation resolution and tissue repair by inhibiting pro-inflammatory transcription factors, including NF-κB and AP-1. In the atopic dermatitis context, *NR4A1* dysfunction may prevent effective inflammation termination, establishing chronic inflammatory states (44, 45). Tamoxifen, as a selective estrogen receptor modulator, may provide estrogen-independent anti-inflammatory therapeutic pathways through *NR4A1* regulation. However, considering tamoxifen’s endocrine effects, its application in atopic dermatitis treatment requires careful benefit-risk assessment, particularly demanding heightened caution in pediatric patients.

### Limitations

While this study provides comprehensive insights into the gut-skin axis mechanisms in atopic dermatitis, several limitations warrant consideration. First, the single-cell RNA sequencing data are limited by sample size (five AD patients and four healthy controls), necessitating validation in larger cohorts to ensure generalizability across different disease severities and clinical phenotypes. Second, our transcriptomic analysis does not directly measure protein abundance or functional activity, and post-transcriptional regulation may significantly influence the biological effects of identified genes. Third, while we established associations between gut microbial metabolites and skin pathology through bioinformatic approaches, direct mechanistic links require further experimental validation using *in vitro* and *in vivo* models. Finally, the *in vitro* validation experiments were conducted using a simplified cell culture model that may not fully recapitulate the complex microenvironment of atopic dermatitis skin.

### Conclusion

This comprehensive study systematically elucidated the molecular mechanisms underlying the "gut-skin axis" in atopic dermatitis through integrated approaches, including single-cell RNA sequencing and computational pharmacology. Our findings establish a causal relationship between specific gut bacteria (*Eubacterium eligens* group) and atopic dermatitis risk while identifying seven key bridging genes (*AKR1C2*, *GALE*, *GGH*, *NR4A1*, *PLA2G4B*, *TYMS*) that connect gut microbial metabolites to skin pathology. Single-cell analysis revealed significant cellular heterogeneity with keratinocytes serving as central coordinators in the inflammatory microenvironment. Notably, molecular docking studies uncovered unexpected high-affinity interactions between methotrexate and *GALE*, surpassing its classical target *TYMS*, suggesting novel therapeutic mechanisms involving glucose metabolism and skin barrier function. These findings collectively establish the gut-skin axis as a critical therapeutic target, offering an evidence-based foundation for developing precision medicine approaches that integrate microbiome interventions with targeted pharmacotherapy for atopic dermatitis management.

## SUPPLEMENTAL MATERIAL

Fig. S1 msystems.01403-25-s0001.tif Flow chart.

Fig. S2 msystems.01403-25-s0002.tiff Reverse Mendelian randomization analysis of coronary heart disease on the *Eubacterium eligens* group.

Fig. S3 msystems.01403-25-s0003.tiff Reverse Mendelian randomization analysis of coronary heart disease on *Sellimonas*.

Legends msystems.01403-25-s0004.docx Supplemental figure and table legends.

Table S1 msystems.01403-25-s0005.xlsx RNA concentration information.

Table S2 msystems.01403-25-s0006.xlsx GALE quantification Cq results.

Table S3 msystems.01403-25-s0007.xlsx Date analysis.

ASM does not own the copyrights to Supplemental Material that may be linked to, or accessed through, an article. The authors have granted ASM a non-exclusive, world-wide license to publish the Supplemental Material files. Please contact the corresponding author directly for reuse.
