# Supplementary figures and images for: Integrative multi-omics analysis reveals gut-skin axis mechanisms and novel therapeutic target GALE in atopic dermatitis
Source: mSystems. 2025 Dec 5;11(1):e01403-25. doi: 10.1128/msystems.01403-25 (PMC12817900; doi:10.1128/msystems.01403-25)

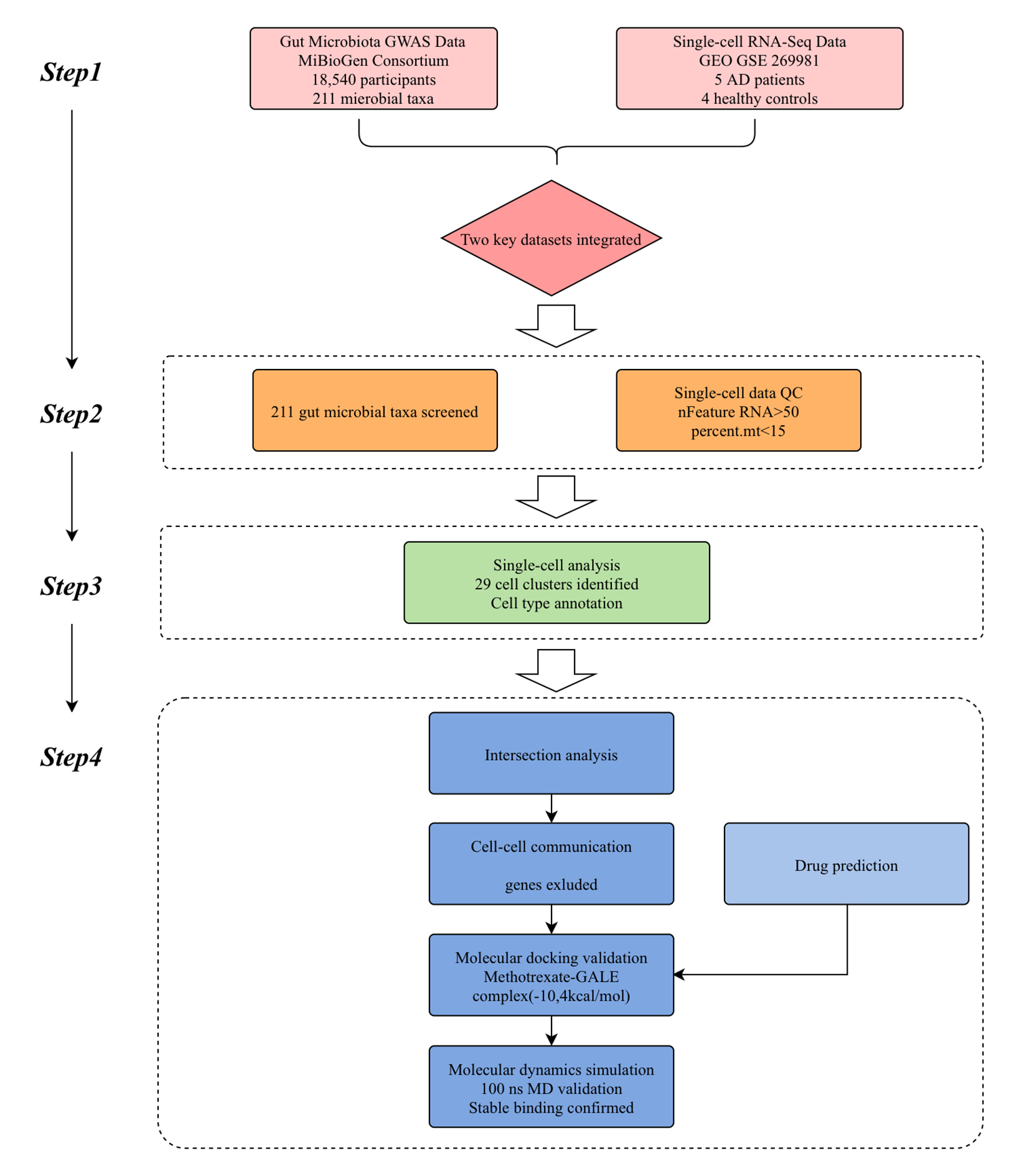

Supplement: Fig. S1 — Flow chart. [file msystems.01403-25-s0001.tif]

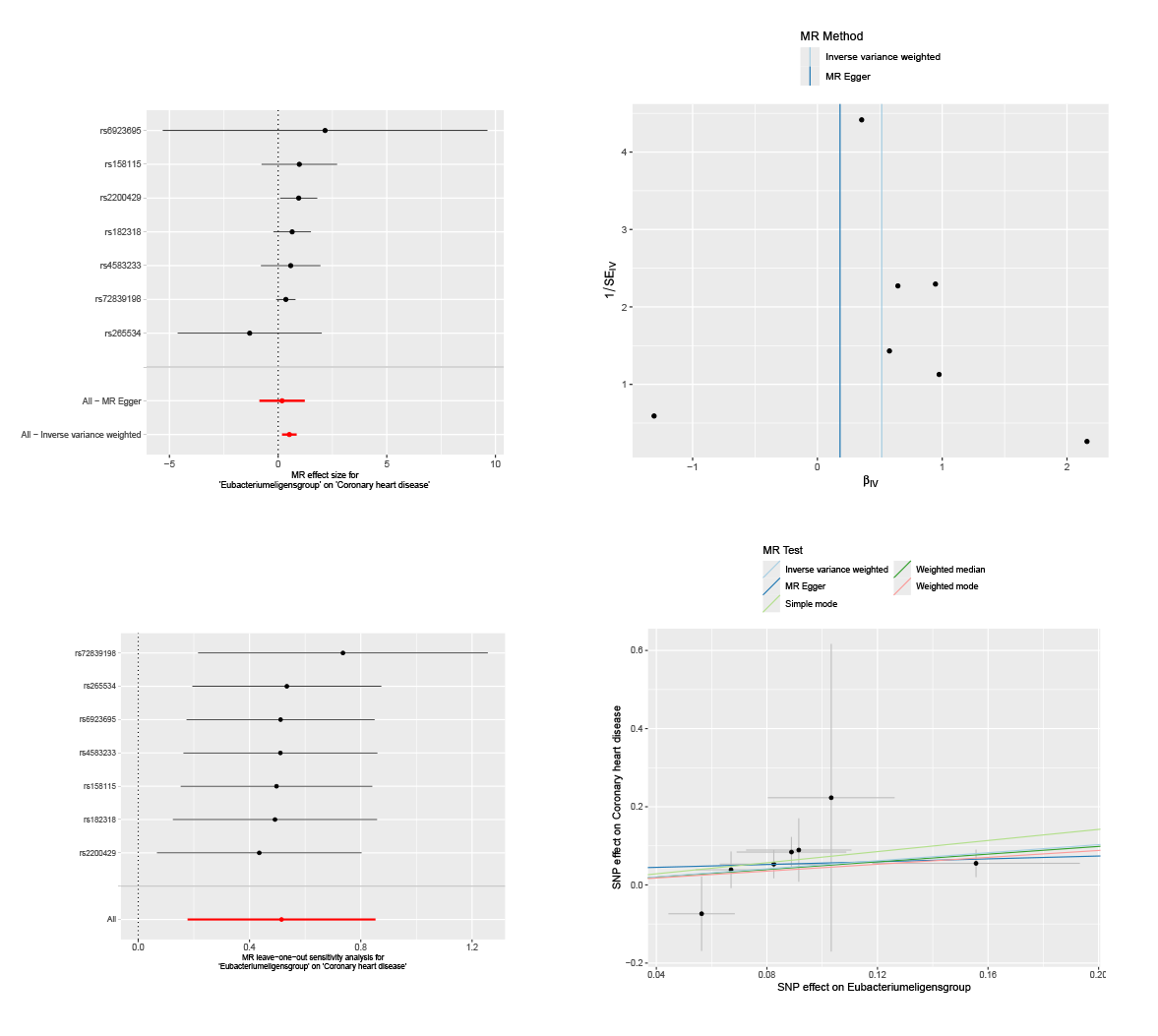

Supplement: Fig. S2 — Reverse Mendelian randomization analysis of coronary heart disease on the Eubacterium eligens group. [file msystems.01403-25-s0002.tiff]

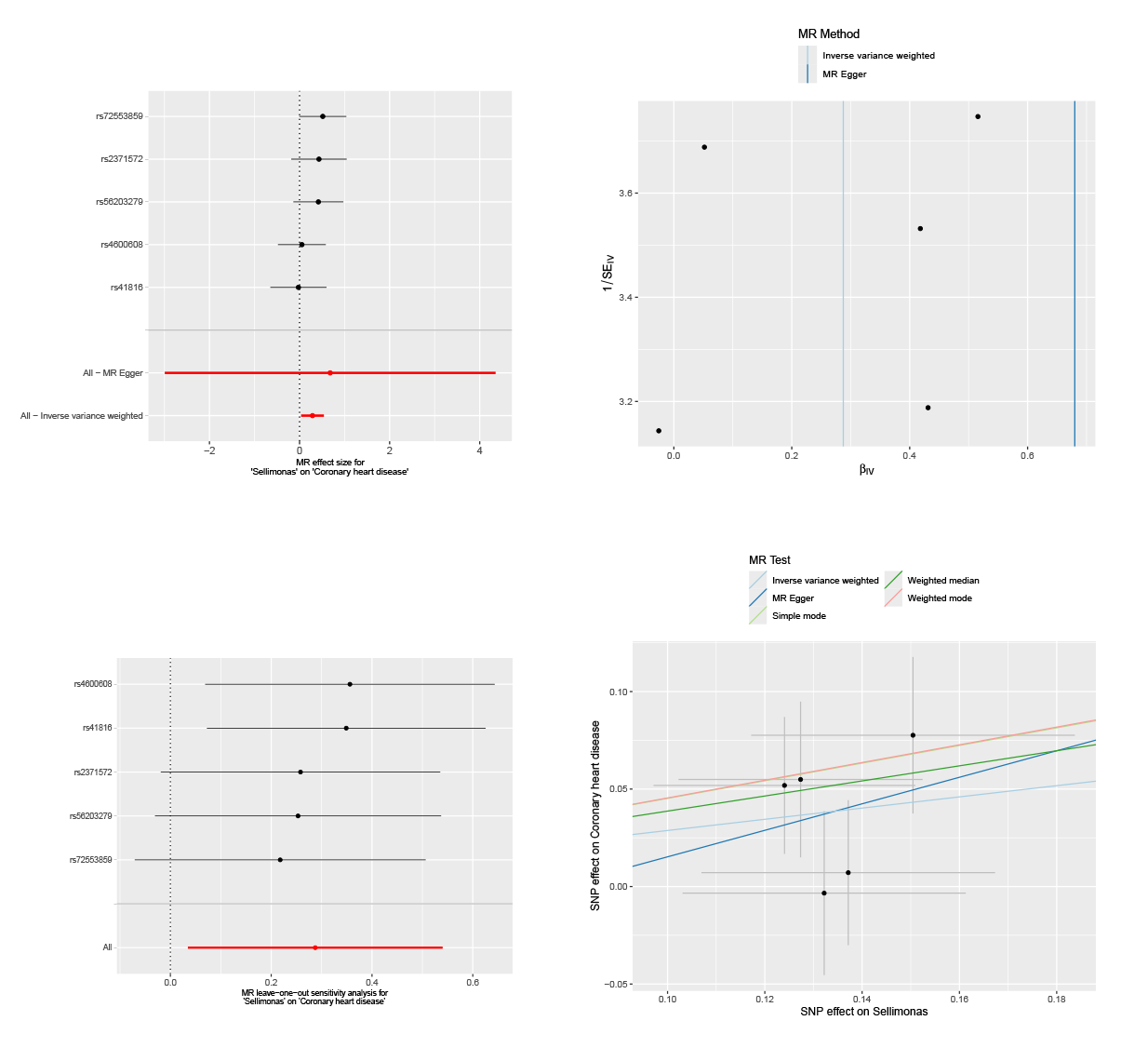

Supplement: Fig. S3 — Reverse Mendelian randomization analysis of coronary heart disease on Sellimonas. [file msystems.01403-25-s0003.tiff]
