# Supplementary material for: Integrative multi-omics analysis reveals gut-skin axis mechanisms and novel therapeutic target GALE in atopic dermatitis
Source: mSystems. 2025 Dec 5;11(1):e01403-25. doi: 10.1128/msystems.01403-25 (PMC12817900; doi:10.1128/msystems.01403-25)
Supplement: Legends — Supplemental figure and table legends. [file msystems.01403-25-s0004.docx]

**Figure S1.Integrative multi-omics workflow for identifying gut microbiota-atopic dermatitis associations and therapeutic targets.** (The study integrated three key datasets: gut microbiota GWAS data from MiBioGen Consortium (18,540 participants, 211 microbial taxa), atopic dermatitis GWAS data from bbj-a.90 dataset (2,885 AD cases, 209,651 controls), and single-cell RNA-seq data from GEO GSE 269981 (5 AD patients, 4 healthy controls). Following data quality control (Step 2), Mendelian randomization analysis identified 2 significant taxa (Eubacterium eligens, Sellimonas), while single-cell analysis revealed 29 cell clusters with cell type annotation (Step 3). Intersection analysis led to cell-cell communication gene exclusion, drug prediction, and molecular validation through docking (Methotrexate-GALE complex, -10.4 kcal/mol) and 100 ns molecular dynamics simulation confirming stable binding (Step 4).)

**Figure S2**. **Reverse Mendelian randomization analysis of coronary heart disease on Eubacterium eligens group.** (Forest plots show MR effect sizes using inverse variance weighted (IVW) and MR Egger methods for individual SNPs and overall estimates (top left). Funnel plot displays SNP effect sizes against standard errors to assess heterogeneity and pleiotropy (top right). Leave-one-out sensitivity analysis demonstrates the robustness of results by iteratively excluding each SNP (bottom left). Scatter plot illustrates the relationship between SNP effects on coronary heart disease and Eubacterium eligens group abundance, with regression lines for different MR methods including IVW, MR Egger, weighted median, and weighted mode approaches (bottom right). The reverse MR analysis investigates whether coronary heart disease causally influences gut microbiota composition, specifically Eubacterium eligens group abundance.)

**Figure S3. Reverse Mendelian randomization analysis of coronary heart disease on Sellimonas.** (Forest plots show MR effect sizes using inverse variance weighted (IVW) and MR Egger methods for individual SNPs and overall estimates (top left). Funnel plot displays SNP effect sizes against standard errors to assess heterogeneity and pleiotropy (top right). Leave-one-out sensitivity analysis demonstrates the robustness of results by iteratively excluding each SNP (bottom left). Scatter plot illustrates the relationship between SNP effects on coronary heart disease and Sellimonas abundance, with regression lines for different MR methods including IVW, MR Egger, weighted median, weighted mode, and simple mode approaches (bottom right). The reverse MR analysis investigates whether coronary heart disease causally influences gut microbiota composition, specifically Sellimonas abundance.)

**Table S1. RNA Concentration information.** (Detailed RNA concentration information, including Nucleic Acid (ng/uL) and reverse transcription RNA concentration (ng/uL))

**Table S2. GALE Quantification Cq Results.** (The specific experimental group divisions and Cq results).

**Table S3. GALE QPCR data analysis.** (GALE expression in the atopic dermatitis model group showed only a modest increase compared to controls (fold change = 1.16, representing a 16% increase). Methotrexate treatment dramatically upregulated GALE expression by 4.04-fold compared to controls (304% increase)).
